# Presystolic Murmur

**Published:** 1889-12-21

**Authors:** 


### PRESYSTOLIC MURMUR.

Dr. Howship Dickinson's lecture on the "so-called presystolic murmur," given in the *Lancet* of October 19, demands attention. That the murmur in question is "presystolic" is the view of the orthodox. It is one of the most characteristic murmurs the heart can produce. In common phase, "it runs up to the first sound." But Dr. Dickinson says it runs up to the latter part of the first sound. It begins softly at the end of the silence which follows the second sound, increases in volume as it goes on, and terminates abruptly with a sharp short noise like a knock or a snap which concludes the systole. Diagrams are given showing this. The character of the sound suggests that it is made by percussion. Its place associates it with the mitral valve, and its time must be that of the closure of the mitral valve. When this sound is heard the mitral valves are thickened and the orifice contracted, so that the valve is able to close, though from its stiffness it is to be presumed that the closure has been delayed.

